# Supports and Barriers in the Talent Development of Socio-Economically Disadvantaged Gifted Students: A Retrospective Narrative Inquiry

**DOI:** 10.3390/jintelligence14050072

**Published:** 2026-05-01

**Authors:** Chia Chao Li

**Affiliations:** Center for Gifted Education Resources in Elementary Schools, National Taiwan Normal University, New Taipei City 236, Taiwan; u9210376@gmail.com

**Keywords:** gifted education, socio-economic disadvantage, talent development, equity, psychosocial skills, resilience

## Abstract

Equity in gifted education remains a persistent international challenge, particularly regarding the “excellence gap” in advanced achievement and long-term attainment. This study investigates the supports and barriers shaping the talent development of socio-economically disadvantaged gifted students in Taiwan. Using a retrospective narrative inquiry, we analyzed the life stories of 25 alumni from the “Bright Minds Award Program,” a long-term initiative providing financial aid, mentorship, and enrichment opportunities for high-ability learners from low-income households. Findings indicate that participants often displayed early academic promise, yet their developmental trajectories were continuously negotiated under structural constraints (limited material and cultural resources, restricted access to domain-specific cultivation, and opportunity gaps across educational transitions) and the psychosocial burden of poverty (shame, stigma management, and identity strain). Drawing on the Actiotope Model of Giftedness, we identify how exogenous educational capital (e.g., scholarships, information brokerage, mentoring networks) and endogenous learning capital (e.g., resilience, self-regulation, goal persistence) interact to stabilize—or destabilize—developmental pathways. A novel contribution is the emergence of “Acting Middle Class” as a coping mechanism through which participants navigated social stigma and the hidden curriculum of elite educational settings. We argue that effective intervention requires not only resource provision but sustained “educational scaffolding” that is psychologically safe and institutionally stigma-sensitive. Implications are discussed for talent development research, school practice, and equity-oriented policy designs aimed at preventing talent attrition and promoting developmental justice.

## 1. Introduction

Equity in gifted education has become one of the most persistent and complex challenges worldwide. Scholars have consistently pointed out the disproportionate representation of students from low-income and marginalized backgrounds in gifted and talented programs (Dixson, 2020; Jung et al., 2022; Worrell & Dixson, 2022). While gifted education seeks to cultivate excellence, opportunities to identify, nurture, and sustain high potential are not equitably distributed. Socio-economic disadvantage, in particular, has been recognized as a major barrier to talent development, restricting access to quality learning experiences, enrichment resources, and long-term academic pathways (Olszewski-Kubilius & Corwith, 2018). Such inequities contribute to what has been termed the “excellence gap” (Dixson, 2022; J. A. Plucker et al., 2017; J. A. Plucker & Peters, 2018; S. J. Peters et al., 2019; S. F. Peters, 2022) in which differences in achievement and advanced attainment are systematically associated with social class and resource allocation rather than potential alone. While a growing body of research has emphasized inequities in gifted education (J. Plucker et al., 2015; J. A. Plucker & Peters, 2016), some studies have also highlighted the effectiveness of targeted interventions in mitigating these disparities, suggesting that structural barriers can be partially addressed through sustained support systems.

A growing body of scholarship has also begun to challenge deficit framings of disadvantaged learners by emphasizing the possibility of “hidden talents” and adaptive competencies developed under harsh or resource-limited environments (S. J. Peters & Engerrand, 2016; Ellis et al., 2020). This perspective suggests that the developmental question is not whether disadvantaged students have ability, but whether educational ecosystems provide the right affordances for that ability to become stable competence and, eventually, expertise. This shift in framing aligns with contemporary talent development perspectives that emphasize dynamic person–environment interactions and the accumulation of domain-relevant opportunities over time (Subotnik et al., 2011, 2019).

In Taiwan, issues related to socio-economically disadvantaged gifted students have received comparatively limited and fragmented scholarly and policy attention. While international research has increasingly emphasized equity concerns in gifted education (e.g., Olszewski-Kubilius & Corwith, 2018; J. A. Plucker & Peters, 2018), related discussions in the Taiwanese context remain relatively underdeveloped and lack systematic empirical investigation. Gifted education in Taiwan is primarily implemented through a formal identification and placement system within the national special education framework. Students are typically identified through multi-stage evaluation procedures, including standardized assessments, academic performance, teacher recommendations, and, in some cases, domain-specific aptitude tests. Identified students may be placed in resource room programs, pull-out enrichment models, or, less commonly, specialized gifted classes, depending on local policy and school-level implementation (Wu & Kuo, 2016). While this system has been effective in identifying students with high academic potential, access to gifted education opportunities remains uneven. Students from middle- and high-socioeconomic backgrounds are more likely to benefit from preparatory resources, parental support, and information about identification pathways. In contrast, students from low-income households often face structural barriers, including limited access to preparatory learning experiences, reduced cultural capital, and lower visibility within school nomination processes. Importantly, Taiwan’s formal gifted education system has not historically incorporated comprehensive or targeted support mechanisms specifically designed for socio-economically disadvantaged gifted students. In response to this gap, supplementary programs—such as the Bright Minds Award Program, jointly established by Morgan Stanley and the Chinese Association for Gifted Education in 2004—have sought to provide sustained support for high-ability students from low-income households. The program offers scholarships, mentorship, and enrichment opportunities aimed at mitigating structural barriers and supporting long-term talent development (Yu et al., 2020). Despite the significance of such initiatives, systematic research on participants’ developmental trajectories—and on how supports and barriers are experienced, interpreted, and negotiated over time—remains limited.

Understanding how disadvantaged gifted students navigate their development requires examining both environmental constraints and enabling factors. Prior studies suggest that psychosocial skills such as resilience, motivation, and self-regulation play critical roles in mediating the effects of poverty (Lipnevich et al., 2016; Subotnik et al., 2019; Rinn, 2020). However, less is known about how these skills are cultivated, at what psychological cost, and how they interact with external supports (teachers, mentors, and off-campus opportunities) in the Taiwanese context. Moreover, equity-oriented support can have unintended consequences if implemented without sensitivity—for instance, identification or aid procedures may inadvertently amplify stigma (Ziegler et al., 2021). This study therefore analyzes the life stories of socio-economically disadvantaged gifted students to identify the supports and barriers shaping their trajectories, contributing an integrative, context-sensitive account to global conversations on talent development and educational justice. Driven by the talent development of gifted students in disadvantaged socio-economic settings, this study aims to: (a) Describe the developmental experiences and talent trajectories of gifted students from socio-economically disadvantaged backgrounds. (b) Analyze the supports and barriers they encountered across critical educational transitions, emphasizing how internal resources and external capital interact over time.

## 2. Theoretical Framework

The study of socio-economically disadvantaged gifted students requires a multidimensional perspective that considers both individual strengths and environmental conditions. Two complementary frameworks—the Talent Development Mega-Model and the Actiotope Model of Giftedness—provide a useful lens for analyzing supports and barriers.

### 2.1. Talent Development Mega-Model

The Talent Development Mega-Model (Olszewski-Kubilius, 2024; Subotnik et al., 2011, 2021, 2023) conceptualizes giftedness as a developmental process that progresses from potential to competence, expertise, and ultimately eminence. This view reframes giftedness as a trajectory that must be actively supported over time, rather than a stable trait. In addition to cognitive ability, the model highlights psychosocial skills (motivation, self-regulation, resilience, and identity-related competencies) as essential for sustaining progress and managing setbacks (Subotnik et al., 2019). Socio-economic disadvantage, within this framework, is not merely a background variable; it is a structuring condition that shapes access to instruction, role models, and opportunity structures. Therefore, interventions that cultivate psychosocial skills and provide sustained mentoring can function as compensatory mechanisms enabling continued advancement.

### 2.2. Actiotope Model of Giftedness

The Actiotope Model of Giftedness (Ziegler & Baker, 2013; Ziegler et al., 2017; Phillipson et al., 2023) emphasizes the reciprocal interaction between individuals and their environments, conceptualizing talent development as adaptation. It distinguishes between exogenous educational capital (resources and affordances provided by the environment, such as financial support, mentoring networks, cultural opportunities, and institutional access) and endogenous learning capital (internal resources, including self-concept, volition, resilience, and actional regulation). From this perspective, poverty can be understood as a systematic depletion of exogenous capital that increases the developmental load placed on endogenous resources. The model’s emphasis on resource configurations is particularly relevant for analyzing disadvantaged contexts, where small changes in exogenous capital (e.g., scholarships or information access) may yield nonlinear effects on trajectories.

### 2.3. Integrative Use of Both Frameworks

Together, these frameworks illuminate how talent development among disadvantaged gifted students depends on (a) the availability and timing of exogenous resources, (b) the development and sustainability of psychosocial capacities, and (c) the degree to which institutional practices either reduce or intensify stigma. In this study, the Mega-Model helps interpret participants’ developmental stages and psychosocial demands, while the Actiotope Model clarifies how educational and learning capital interact to stabilize—or destabilize—talent trajectories. While both models offer powerful frameworks for understanding talent development, they also have limitations. The Talent Development Mega-Model has been critiqued for its emphasis on linear progression and high-level achievement, which may not fully capture the non-linear and context-dependent trajectories of individuals from disadvantaged backgrounds. Similarly, the Actiotope Model, while comprehensive in its consideration of environmental and internal resources, may be analytically complex and challenging to operationalize in empirical research.

Despite these limitations, both models provide valuable conceptual tools for examining the dynamic interaction between individual capacities and contextual conditions, particularly in relation to equity and access in gifted education.

## 3. Method

In this study, a retrospective narrative inquiry was used. Researchers described the talent development of these gifted students from socio-economically disadvantaged backgrounds. Through the narrative interview method, the researchers analyzed and summarized the talent development of each individual and explore the support and resistance of their talent development. As such, the narrative interview method was chosen as the research methodology. In this study, the targeted participants were the students from the Bright Minds Award Program who agreed to participate in the study.

Through a retrospective narrative inquiry, the study explored the process and experience of each individual’s talent development. Since every student was different due to his or her family environment and situations, the processes of talent development of each one differed from others as well. Through reminiscing and genuine understanding, the research tried to collect the life experiences of the participants. To fully understand the experiences of gifted students from disadvantaged socio-economic backgrounds, the study had to commence from each participant’s perspective.

### 3.1. Participants

The participants in this study were 25 alumni of the Bright Minds Award Program, a long-term initiative established in Taiwan to support gifted students from socio-economically disadvantaged families. The program initially served junior high school students who met government criteria for low-income households and demonstrated high academic potential. At the time of data collection, participants ranged in age from 20 to 30 years, representing diverse trajectories in higher education and early career development.

Participants were recruited from the 157 program recipients across six cohorts using purposeful and snowball sampling. Prior to participation, all participants were fully informed of the purpose, procedures, and voluntary nature of the study, and written informed consent was obtained before the interviews were conducted. Participation was entirely voluntary, and participants were informed of their right to withdraw from the study at any time without penalty.

To gain an in-depth understanding of the talent development trajectories of socio-economically disadvantaged high-achieving students, this study considered the diversity of participants’ educational and career pathways. Alumni were invited—via the program’s LINE group chats and its Facebook community—to share their talent development trajectories and life stories. In total, 25 alumni participated in this study, representing multiple cohorts and a wide range of educational and occupational fields. Table 1 presents the talent development domains and background information of the 25 participants. The distribution of participants across cohorts reflects both availability and willingness to participate, as well as accessibility through program communication networks. As such, certain cohorts are more represented than others, which may influence the range of experiences captured in this study.

### 3.2. Data Collection

This study adopts narrative inquiry as an overarching methodological framework (Connelly & Clandinin, 1990). Within this framework, autobiographical narrative interviews were conducted following Schütze’s (2011) approach to elicit participants’ life stories across time, context, and social conditions.

Narrative interviews enable participants to provide detailed accounts of personal life events and developmental trajectories within the boundaries of the research focus. Through dialogic interaction between the interviewer and participants, rich life-history data were generated, capturing lived experiences over time. Data collection was conducted between February 2020 and March 2021. A total of 25 participants were interviewed, each participating in an in-depth interview lasting approximately 2 to 3 h.

A central principle of Schütze’s narrative interview method is minimizing researcher interference during the storytelling process. Participants are encouraged to narrate their experiences freely, without being constrained by a predetermined interview structure or influenced by example stories. Each interview began with a single open-ended prompt designed to initiate spontaneous narration. Participants were invited to recount their life stories from childhood to the present, including family background, educational experiences, and work trajectories. This approach preserves participants’ autonomy in structuring their accounts while ensuring relevance to the research focus.

During narration, participants’ storytelling is shaped by internal psychological processes such as detailing, gestalt closure, and condensation, as they reconstruct and organize past experiences. Following Schütze’s framework, the narrative interview process consisted of three stages:

#### 3.2.1. Initiation Stage

The interview began with an open-ended narrative question: “Can you please tell me about your life journey, starting from your earliest memories in childhood through adolescence and into the present, including your family background, educational experiences, and work history?” During this stage, the researcher adopted the role of an attentive listener and refrained from interrupting, allowing participants to narrate freely until they indicated completion.

#### 3.2.2. Questioning Stage

In the second stage, follow-up questions were posed to clarify ambiguities, address interruptions in the narrative flow, and invite elaboration on briefly mentioned or omitted experiences. This process enhanced the completeness and coherence of participants’ narratives.

#### 3.2.3. Balancing Stage

The final stage encouraged participants to engage in reflective evaluation of their life narratives. Through guided prompts, participants were invited to interpret their own experiences and developmental trajectories.

All interviews were audio-recorded with informed consent and transcribed verbatim. Identifying information (e.g., participant codes, dates, and locations) was systematically documented. The resulting life narratives captured longitudinal developmental trajectories across family, educational, and early career contexts, providing a rich empirical basis for subsequent analysis.

### 3.3. Data Analysis

Data analysis was conducted following the completion of all interviews. Within the narrative inquiry framework, multiple analytic strategies were employed for complementary purposes. Structural narrative analysis was used to reconstruct developmental sequences and turning points across participants’ life trajectories. Linguistic indicator analysis was not treated as a separate methodological approach, but as an analytic lens embedded within the narrative inquiry framework. Specifically, it focused on how participants linguistically constructed and expressed their experiences through evaluative language, emotional expressions, and identity-related positioning. Through this process, patterns of meaning-making—such as stigma management, resilience, and identity negotiation—were identified and interpreted in relation to participants’ developmental trajectories.

Consistent with narrative inquiry traditions, the analysis was guided by three dimensions: temporality (changes and continuities over time), sociality (personal and social conditions shaping experience), and place (the contextual settings in which experiences occur) (Connelly & Clandinin, 1990). The analysis was conducted manually to allow for close interpretive engagement with participants’ narratives, consistent with qualitative traditions that emphasize depth and contextual understanding.

The analytic process followed a three-stage procedure adapted from Schütze’s (2011) framework:

#### 3.3.1. Basic Structural Framing of Narrative Texts

Each transcript was segmented into analytic units by distinguishing narrative passages from non-narrative content (e.g., background explanations or argumentative statements). Non-narrative sections were temporarily set aside to preserve the integrity of the narrative flow.

#### 3.3.2. Structural Description of Narrative Action Sequences

Narrative passages were analyzed sequentially to reconstruct the action structures underlying participants’ experiences. This process focused on identifying turning points, decision-making moments, and patterns of action that revealed the internal coherence of developmental trajectories.

#### 3.3.3. Overall Narrative Shaping and Conceptualization

The final stage involved interpretive synthesis. Individual narratives were examined holistically to identify the social, cultural, and psychological meanings underlying participants’ actions. This process enabled cross-case comparison and theoretical interpretation of talent development under conditions of socioeconomic disadvantage.

To ensure trustworthiness, this study followed Lincoln and Guba’s (1985) criteria of credibility, transferability, dependability, and confirmability. Multiple strategies were employed. First, interviews were audio-recorded and transcribed verbatim, followed by member checking to ensure accuracy and authenticity. Second, a research diary was maintained to document methodological decisions and analytic reflections, thereby providing an audit trail. Third, data and theoretical triangulation were conducted by interpreting participants’ narratives in relation to the existing literature and theoretical frameworks, thereby enhancing analytic rigor and consistency.

Together, these procedures ensured that the findings were grounded in participants’ lived experiences, systematically documented, and analytically coherent.

## 4. Results

The current literature on talent development often prioritizes individuals who have already attained peak performance or eminence in their respective fields (Subotnik et al., 2019). However, because the participants in this study were primarily in their early adulthood (aged 20 to 30), their trajectories are characterized by emerging competence rather than fully realized eminence. From the perspective of the Actiotope Model of Giftedness (Ziegler & Baker, 2013; Ziegler et al., 2017), their developmental journeys highlight the critical interplay of “learning resources”—specifically exogenous educational capital and endogenous learning capital—in navigating adversities. The following sections detail the specific supports and barriers encountered by these socio-economically disadvantaged gifted students. The following themes were derived through cross-case comparative analysis, informed by narrative inquiry, structural narrative analysis, and linguistic indicator analysis. Rather than representing isolated individual accounts, the findings reflect recurring patterns identified across participants’ developmental trajectories. These themes highlight shared mechanisms through which supports and barriers operate, while also preserving the contextual richness of individual life stories.

### 4.1. Supports

Supports acted as vital exogenous and endogenous resources that facilitated the talent development of socio-economically disadvantaged gifted students, compensating for the deficiencies in their immediate environments.

#### 4.1.1. Teacher Support

Across participants’ narratives, teachers consistently emerged as a critical source of support. Structural analysis of narrative sequences revealed that teacher intervention often occurred at key developmental turning points, particularly during early schooling stages when family support was limited. Teachers played a pivotal role in compensating for limited familial support, particularly during participants’ early developmental stages. For some students, teachers were not only academic instructors but also the primary adults who recognized their emotional needs and personal struggles. One participant (S19) described how her strong academic effort in elementary school was driven by a desire to gain her father’s attention—an effort that, despite consistent academic success, failed to result in parental recognition. This family context significantly shaped her emotional development and self-concept. During this period, elementary school teachers—especially those in the lower and middle grades—became the individuals who understood her best. Because these teachers were aware of her family background, they responded to her behaviors with empathy rather than punishment, providing emotional containment and acceptance even when she exhibited behavioral difficulties. Such teacher responses functioned as a form of emotional scaffolding, offering stability and affirmation at a time when support from home was limited. As S19 reflected, the tolerance, understanding, and care shown by her teachers during this challenging period left a lasting impact on her development and remained a source of gratitude in adulthood.

“…During that really difficult period of my life, I am very grateful to the school teachers who took good care of me. There were some teachers who had a profound influence on my life… The teachers who taught me in third and fourth grade took especially good care of me. They knew about my family situation, so even when I behaved badly at school… they would not punish me… This teacher was always very tolerant of me and treated me kindly. I still feel deeply grateful for how well this teacher treated me.”

#### 4.1.2. Off-Campus Learning Opportunities

Participation in enrichment activities, camps, and mentorship programs broadened students’ horizons and allowed them to interact with peers from similar backgrounds. These experiences expanded their social and cultural capital, providing exposure to diverse educational and career pathways.

A prime example of this exogenous educational capital (Ziegler et al., 2017) was the Bright Minds Award Program. The majority of participants emphasized how the program’s financial assistance and networking opportunities became crucial turning points. S21 highlighted the emotional and social bonds formed:

“Essentially, the help is the scholarship, and maybe the established emotional bonds. Even if you don’t regularly meet or stay in touch with those people, you exist because we were all a part of the Bright Minds Program… The program made me broaden the perspectives.”

Furthermore, these off-campus programs provided didactic and cultural capital by reducing information asymmetry regarding university admissions. S12 recounted how the program offered a psychologically safe space:

“The topic of how to apply for the senior high school has helped me a lot… There were very few applicants applying through this channel because you usually won’t tell others about your family background. It makes me feel very embarrassed… In the Bright Minds Awards Project, everyone’s background should be similar… so there’s no such big pressure in discussing this topic. I learned a lot of things here.”

#### 4.1.3. Psychosocial Skills

Linguistic indicators within participants’ narratives—such as expressions of persistence, emotional struggle, and self-regulation—further highlighted the central role of psychosocial skills as coping resources under conditions of socioeconomic strain. Across cases, three psychosocial skills—resilience, motivation, and self-regulation—emerged as particularly salient forms of endogenous learning capital that sustained talent development under conditions of socioeconomic strain (Worrell et al., 2019). Rather than functioning as abstract personality traits, these skills were narrated as practical coping resources cultivated through repeated encounters with academic pressure, limited family resources, and critical educational transitions.

While these coping mechanisms enabled participants to persist and succeed in the short term, they may also carry long-term implications. For example, sustained emotional suppression, heightened self-reliance, and identity management may influence career choices, interpersonal relationships, and self-concept development in adulthood. These patterns suggest that the psychological costs of adaptation may extend beyond schooling and continue to shape individuals’ long-term developmental trajectories.

##### Resilience

Participants’ narratives revealed resilience as the capacity to persist under prolonged stress and to recalibrate after setbacks, rather than as simple endurance. For example, S13 recalled experiencing intense academic pressure and confusion during high school, yet continuing to strive rather than disengage:

“…From tenth to twelfth grade, I never gave up on myself. I worked very, very hard, but it felt like I couldn’t ‘get it.’ When the amount of content kept increasing, I completely fell into chaos…”

In other cases, resilience involved recognizing personal limits and deliberately slowing down to prevent further harm. S17 described reducing academic pace in graduate school after serious health problems emerged:

“…After entering graduate school, I slowed down… I couldn’t keep going the way I used to because my body had too many problems… so graduate school became mainly about recovering and writing my thesis gradually…”

From an actiotope perspective, resilience functioned as a protective endogenous resource that allowed individuals to maintain developmental continuity despite chronic external deficits.

##### Motivation

Motivation appeared as a forward-driving force that helped participants maintain direction and purpose, often anchored in concrete goals such as economic stability or meaningful work. S23 illustrated goal-oriented motivation by describing an early decision to pursue stable employment soon after entering university:

“…I decided to take the civil service exam… I hoped I could work right after graduation… so later I chose to apply for a position in a state-owned bank…”

Intrinsic motivation was particularly salient among participants in helping professions. S5 emphasized that social work aligned with personal values and sustained long-term engagement:

“…I’m not ambitious about making money… That’s not my passion. Social work is my passion—being a helping professional is what I feel is meaningful.”

Motivation was further reinforced through experiences of competence and affirmation. S13 described how recognition in university strengthened self-efficacy and sustained aspiration:

“…In university I felt like a fish in water. I know what I want to do, I do my best, and people affirm me… I can see opportunities behind what I do—this cycle keeps showing me what kind of role I can play in society…”

Consistent with the Talent Development Mega-Model, motivation operated as a developmental engine that transformed potential into sustained engagement when environmental support was fragile.

##### Self-Regulation

Self-regulation emerged as a critical skill for managing time, effort, and emotional strain, particularly when participants balanced academic demands with financial necessity. S2 described a highly disciplined daily routine while preparing for graduate entrance and professional licensure examinations:

“…My study routine was: wake up at 7 a.m., study in the library until 10 or 11 p.m.… for almost a year it was this cycle—first preparing for graduate school entrance exams, then preparing for the bar exam.”

S17 similarly highlighted the intensity of preparation for national examinations and the necessity of extended study hours:

“…The national exam pressure was huge… we had to take 17 subjects… I remember we studied about 11 h a day…”

Self-regulation also involved emotional management when anxiety became overwhelming. S22 described seeking medical support and learning to stabilize anxiety in order to sustain daily functioning and creative work:

“…I went to see a doctor… I had severe anxiety… later psychiatry showed it was psychological pain causing bodily changes, and I started taking medication…”

Within the Actiotope Model, self-regulation represents a core endogenous learning capital that enables individuals to allocate limited internal resources strategically in response to persistent environmental demands.

### 4.2. Barriers

Despite the presence of supports, participants’ talent trajectories were frequently constrained by structural and psychological barriers that taxed their developmental energy.

#### 4.2.1. Family Resource Limitations

Poverty, single-parent households, and limited parental education restricted access to material, cultural, and emotional support. Participants frequently described experiences of financial insecurity and the lack of opportunities to pursue specialized training.

This lack of resources often manifested as a profound sense of “relative deprivation” when participants entered elite higher education environments. S17, who navigated her way from a remote mountainous area to National Taiwan University (NTU), articulated this gap in holistic educational capital:

“I feel that I have done a good job from what I’ve got. Well, of course, I think about that if I were born into a family with a good background with a lot of resources, would I have become better?… I may not be much different from them [my classmates at NTU] academically, but they are very good in many other things. Like, my English is actually really bad… But a lot of my classmates lived abroad for a few years… and they learned a bunch of things like playing the piano.”

#### 4.2.2. Feelings of Inferiority and Social Stigma

Many participants reported shame or embarrassment associated with their low-income status. This stigma affected their self-concept and peer relationships, often leading to social withdrawal or the concealment of their economic background.

Drawing a parallel to Fordham and Ogbu’s (1986) concept of “acting white”—where marginalized students fear being alienated by their peers for academic success—this study identified a phenomenon of “Acting Middle Class.” Participants often concealed their poverty to avoid judgment. S07 reflected on the psychological toll of this concealment:

“When I was in high school, my self-image was quite bad. I was lacking a lot of self-confidence, and I felt guilty of myself, and I felt that this affected me quite a bit in terms of how I got along with my classmates… I dare not talk and interact with my classmates.”

Furthermore, institutional insensitivity often exacerbated this stigma. S14 described the humiliation of being publicly identified as a low-income student, illustrating how administrative processes can damage a student’s fragile self-concept:

“I didn’t want to use it [the low-income status]. I felt very ashamed… But one time… the school had broadcasted the names of all the low-income households for the whole school. I found out that, except for me, the other students were all bad students from the school. And I just felt very ashamed and humiliated, so I didn’t want to go.”

#### 4.2.3. Sacrifices for Family Obligations

A recurring theme across participants’ narratives was the pressure to prioritize family needs over personal aspirations. Several participants assumed adult responsibilities at an early age, such as caring for siblings or contributing financially to household stability. These early role expectations often interrupted or delayed talent development, as individuals were compelled to allocate their limited endogenous developmental energy toward family survival rather than sustained academic exploration or specialization.

Rather than framing S23’s decisions as deliberate long-term “planning” for individual advancement, her narrative reflects a form of family-centered striving. She described her educational and career choices as driven by a desire to protect her mother—who had worked tirelessly for much of her life—from continued hardship and worry. For S23, life decisions were not oriented primarily toward personal fulfillment, but toward fulfilling familial responsibility and ensuring collective well-being. As S23 explained:

“Actually, I don’t think of it as ‘planning.’ I feel that I’m not just an individual—I am a family. My mother has already worked so hard during the first half of her life. Now that we’ve grown up, we should be the ones working. I don’t want my mother to keep struggling the way she does now when I grow up. So I felt that once I graduated, I had to have a job, because my mother was already getting older…”

From a talent development perspective, such family-oriented decision-making can be understood as a form of moral obligation and early role assumption, in which endogenous developmental energy is redirected from individual exploration toward family stability. While this orientation may cultivate maturity, responsibility, and perseverance, it can simultaneously constrain opportunities for prolonged exploration, risk-taking, and domain-specific specialization. As a result, the pace and scope of talent development are shaped not only by individual capacity, but also by the ethical and relational demands embedded within students’ familial contexts.

## 5. Discussion

The interpretations presented in this section are grounded in the thematic patterns identified through cross-case analysis, informed by narrative inquiry, structural narrative analysis, and linguistic indicator analysis. The themes discussed below reflect recurring patterns observed across participants’ developmental trajectories, rather than isolated individual accounts.

This study provides a nuanced account of how socio-economically disadvantaged gifted students in Taiwan navigate their talent development through a complex interplay of supports and barriers. Rather than portraying talent development as a linear progression, the findings reveal a fragile and resource-dependent process, shaped by the continuous negotiation between external opportunities and internal coping mechanisms. By integrating the Talent Development Mega-Model and the Actiotope Model of Giftedness, this discussion advances current understandings of equity in gifted education in three key ways: (a) by reframing supports as forms of educational scaffolding rather than simple resource provision, (b) by conceptualizing psychosocial skills as both assets and adaptive costs, and (c) by foregrounding socio-economic stigma as a central yet under-theorized constraint on gifted identity formation.

### 5.1. Supports as Educational Scaffolding Rather than Substitutes

Consistent with the international literature, teachers and off-campus enrichment programs emerged as pivotal supports for socio-economically disadvantaged gifted students (Horn, 2015; Kaul et al., 2016). However, the present findings suggest that these supports function not merely as compensatory resources, but as developmental scaffolding that temporarily stabilizes otherwise fragile learning environments. Within the Actiotope framework, such supports can be understood as targeted infusions of exogenous educational capital that prevent talent trajectories from collapsing under structural strain.

Teachers, in particular, played a hybrid role that extended beyond instruction to include advocacy, emotional containment, and institutional navigation. For many participants, teachers acted as brokers of opportunity—translating opaque systems of scholarships, examinations, and admissions into actionable pathways. This finding underscores that for disadvantaged gifted students, access alone is insufficient; interpretive guidance is equally critical. Without such mediation, available resources may remain inaccessible or psychologically threatening.

Similarly, programs such as the Bright Minds Award Program did more than alleviate financial burden. They created socially safe micro-contexts in which participants could temporarily suspend the stigma associated with poverty and engage in future-oriented thinking. This aligns with the Talent Development Mega-Model’s emphasis on sustained environmental support, but also extends it by highlighting the emotional safety required for gifted students to envision long-term trajectories. In this sense, off-campus programs served as identity-affirming spaces that counteracted deficit-based narratives prevalent in students’ everyday school lives.

### 5.2. Psychosocial Skills as Double-Edged Endogenous Capital

These findings are consistent with previous research highlighting the role of psychosocial skills in talent development under conditions of adversity (e.g., Subotnik et al., 2019; Ellis et al., 2020). Psychosocial skills—resilience, perseverance, and self-regulation—were repeatedly identified as crucial endogenous learning capital enabling participants to persist despite adversity. These findings strongly support the Talent Development Mega-Model’s assertion that psychosocial competencies are indispensable for transforming potential into sustained competence (Subotnik et al., 2019). However, the narratives also reveal a critical tension: the very skills that facilitate survival and success may exact long-term psychological costs.

Many participants developed heightened self-discipline, emotional suppression, and hyper-responsibility at an early age, often in response to family instability or economic precarity. While these traits supported academic persistence, they were frequently accompanied by chronic self-doubt, guilt, and fear of failure. From an actiotope perspective, this suggests that endogenous capital was accumulated under conditions of scarcity, requiring students to over-invest internal resources to compensate for persistent external deficits.

This finding complicates dominant meritocratic narratives that celebrate resilience without interrogating its origins or consequences. For disadvantaged gifted students, resilience was less a freely cultivated strength than a necessary adaptation to structural neglect. Thus, psychosocial skills should not be romanticized as universal solutions; instead, they must be understood as contextually induced strategies whose sustainability depends on concurrent improvements in exogenous educational capital.

### 5.3. “Acting Middle Class” and the Hidden Curriculum of Belonging

One of the most theoretically significant contributions of this study is the identification of “Acting Middle Class” as a recurring coping mechanism among socio-economically disadvantaged gifted students. Echoing but distinct from Fordham and Ogbu’s (1986) concept of “acting white,” this phenomenon reflects students’ efforts to conceal poverty and perform normative middle-class dispositions in order to gain social acceptance and institutional legitimacy.

Unlike resistance-based interpretations of identity conflict, “Acting Middle Class” in this study functioned as a form of strategic conformity. Participants actively learned the hidden curriculum of elite educational spaces—language use, cultural references, extracurricular norms—while simultaneously suppressing aspects of their lived experience. This identity work demanded constant vigilance and emotional labor, often resulting in social withdrawal and internalized inferiority.

Importantly, institutional practices sometimes intensified this burden. Administratively insensitive procedures, such as the public disclosure of low-income status, transformed structural support mechanisms into sources of symbolic violence. These findings highlight that equity-oriented policies can inadvertently reproduce stigma when implemented without psychosocial awareness. From a systems perspective, such practices represent a misalignment between policy intent and actiotope functioning, undermining the very endogenous resources—self-concept and motivation—that gifted education seeks to cultivate.

### 5.4. Talent Development Under Conditions of Moral Obligation

A further barrier that warrants deeper consideration is the moral weight of family obligation. Many participants experienced forms of parentification, assuming caregiving or financial responsibilities that diverted time, energy, and emotional capacity away from talent development. This finding resonates with ecological models of development but is rarely foregrounded in gifted education research.

Within the Talent Development Mega-Model, such obligations can be understood as competing developmental demands that interrupt the progression from competence to expertise. More critically, they reveal how socio-economic disadvantage embeds gifted students within relational economies of survival, where personal aspiration is morally negotiated rather than individually pursued. Talent development, in this context, becomes an ethical balancing act rather than a purely educational endeavor.

This study examined the developmental trajectories of socio-economically disadvantaged gifted students in Taiwan, identifying both supports and barriers that shaped their talent development. The findings align with international research emphasizing the importance of psychosocial skills and contextual resources in navigating inequities in gifted education (Olszewski-Kubilius & Corwith, 2018; Subotnik et al., 2011).

Taken together, the findings suggest that talent development for socio-economically disadvantaged gifted students is not simply a matter of providing more resources, but of reconfiguring developmental ecosystems. Supports must be sustained, psychosocially attuned, and institutionally sensitive. Without such alignment, endogenous strengths may be depleted rather than cultivated, and external interventions may yield only fragile gains.

By foregrounding lived experiences and identity negotiations, this study extends both the Actiotope Model and the Talent Development Mega-Model into socio-culturally complex terrains. It argues for a shift from deficit remediation toward developmental justice—an approach that recognizes talent not as an individual possession, but as a socially contingent achievement requiring collective responsibility.

Despite its contributions, this study has several limitations that should be acknowledged. First, the study is based on retrospective narrative accounts from a relatively small and purposively selected sample of program participants. While this approach allows for in-depth exploration of developmental experiences, the findings are not intended to be generalizable to all socio-economically disadvantaged gifted students. Second, although participants represented diverse developmental domains and backgrounds, this study did not conduct systematic subgroup analyses based on gender, disciplinary fields, or age differences. As a result, potential variations across these dimensions were not fully examined and should be explored in future research. Third, while multiple qualitative analytic strategies were employed to enhance interpretive depth, the integration of narrative, structural, and linguistic analyses may still be subject to researcher interpretation. Future studies could benefit from triangulating qualitative findings with quantitative or longitudinal data to further strengthen analytic rigor. Finally, the study focuses on participants within a specific support program in Taiwan. Although this context provides valuable insights into talent development under socio-economic constraints, the transferability of the findings to other cultural or policy contexts should be considered with caution.

## 6. Implications

The findings of this study carry important implications for research on intelligence, gifted education practice, and educational policy, particularly in contexts marked by socioeconomic inequality. By illuminating how cognitive, emotional, and sociocultural resources interact over time, this study underscores the necessity of adopting a multidimensional and developmentally sensitive perspective on intelligence and talent development.

### 6.1. Implications for Intelligence and Talent Development Research

From a research perspective, this study reinforces the argument that intelligence cannot be fully understood through cognitive ability alone. While participants demonstrated strong academic potential, their developmental trajectories were profoundly shaped by emotional regulation, identity negotiation, and access to sociocultural resources. These findings align with contemporary calls in intelligence research to move beyond unitary IQ frameworks toward integrative models incorporating emotional, motivational, and contextual determinants.

The identification of Acting Middle Class as an adaptive strategy suggests a need to further examine socioeconomic identity management as an underexplored dimension of intelligence-related functioning. This phenomenon reflects not only emotional intelligence—through heightened social awareness and self-regulation—but also creative and cultural intelligence, as students strategically navigate institutional norms and expectations. Future research could investigate how such adaptive strategies influence long-term well-being, creative expression, and the sustainability of talent development.

Methodologically, the use of retrospective narrative inquiry highlights the value of qualitative approaches in intelligence research. Life-story data provide insights into developmental timing, trade-offs, and cumulative psychological costs that are often invisible in cross-sectional or test-based studies. Longitudinal and mixed-methods research is therefore recommended to trace how IQ-, EQ-, and CQ-related resources interact across life stages, particularly for individuals from disadvantaged backgrounds.

### 6.2. Implications for Gifted Education Practice

For educational practice, the findings suggest that effective support for socio-economically disadvantaged gifted students must extend beyond academic acceleration to include emotionally and culturally responsive interventions. Teachers’ roles as mentors, advocates, and interpreters of institutional systems were central to students’ persistence. This underscores the need for professional development that equips educators with competencies in recognizing hidden giftedness, understanding poverty-related stress, and fostering psychologically safe learning environments.

Moreover, enrichment programs and scholarships should be intentionally designed as identity-affirming spaces, rather than solely as mechanisms for academic enhancement. Providing safe contexts in which students can openly discuss socioeconomic challenges without fear of stigma is critical for sustaining motivation and self-concept. Explicit instruction in psychosocial skills—such as emotional regulation, self-advocacy, and long-term goal setting—can further support students in managing the dual demands of excellence and adversity.

Importantly, this study cautions against over-reliance on individual resilience. While psychosocial skills enabled persistence, they often emerged from necessity rather than choice and were associated with emotional strain. Gifted education programs should therefore balance the cultivation of internal strengths with structural supports that reduce the need for excessive self-compensation.

### 6.3. Implications for Educational Policy and Systems

At the policy level, the findings highlight the importance of systemic sensitivity in equity-oriented initiatives. Financial aid and identification mechanisms intended to support disadvantaged students can inadvertently reinforce stigma when implemented without consideration of students’ emotional and social experiences. Policies should prioritize confidentiality, dignity, and autonomy, ensuring that access to resources does not come at the cost of self-worth.

Sustained, long-term support is also critical. Talent development is cumulative, and short-term interventions are insufficient to counteract enduring socioeconomic constraints. Policymakers should consider multi-year funding structures, mentorship continuity, and cross-institutional coordination to provide stable developmental ecosystems for disadvantaged gifted learners.

Finally, the results call for a shift from deficit-based remediation toward developmental justice. Rather than asking how disadvantaged students can adapt to existing systems, educational policies should examine how systems themselves can be restructured to accommodate diverse developmental pathways. By aligning cognitive enrichment with emotional support and sociocultural inclusion, policymakers can better ensure that high potential is not only identified but meaningfully realized. For educators, this may involve explicitly teaching psychosocial skills such as self-regulation and help-seeking strategies, while also creating classroom environments that are psychologically safe and responsive to students’ socio-economic contexts. Schools can implement mentoring systems and provide structured guidance on academic pathways. At the policy level, efforts should focus on ensuring confidentiality in financial aid processes, sustaining long-term support programs, and integrating psychosocial considerations into gifted education policies.

## 7. Conclusions

This study examined the talent development trajectories of socio-economically disadvantaged gifted students in Taiwan, revealing how developmental outcomes are shaped through the continuous interaction between structural constraints and compensatory supports. The findings demonstrate that limited family resources, social stigma, and early family obligations impose persistent pressures that constrain opportunities for cognitive, emotional, and creative growth. At the same time, supportive teachers, access to off-campus learning opportunities, and the development of psychosocial skills functioned as critical mechanisms that enabled participants to sustain engagement and aspiration despite adversity.

Importantly, the results reinforce the view that talent development is not a linear or purely ability-driven process, but a dynamic and context-dependent system shaped by the alignment—or misalignment—between environmental resources and individual capacities. Consistent with the Talent Development Mega-Model and the Actiotope Model of Giftedness, the study illustrates how exogenous educational capital (e.g., mentorship, financial aid, enrichment opportunities) and endogenous learning capital (e.g., resilience, self-regulation, identity negotiation) jointly influence whether high potential can be maintained over time. These findings caution against approaches that emphasize individual effort alone, highlighting instead the cumulative effects of sustained support and structural sensitivity.

From a policy and practice perspective, the study underscores the need for systematic and long-term interventions to address equity gaps in gifted education. Schools and educational authorities should move beyond episodic support toward coherent support structures that integrate academic enrichment, psychosocial skill development, and culturally sensitive administrative practices. Financial assistance, when combined with mentoring and guidance across educational transitions, is essential to prevent talent attrition and to support the continuity of developmental trajectories.

For future research, the findings point to the value of longitudinal and mixed-methods approaches that can capture how cognitive, emotional, and sociocultural resources interact across life stages. Such research is particularly needed to examine the sustainability of psychosocial adaptations developed under socioeconomic strain and their implications for long-term well-being and achievement.

Ultimately, promoting equity in gifted education requires a shift from deficit-oriented remediation to developmental justice—an approach that recognizes talent as a socially situated potential requiring collective responsibility. By aligning systemic resources with the psychosocial needs of learners, educators and policymakers can create conditions in which socio-economically disadvantaged gifted students are not merely resilient survivors, but individuals empowered to fully realize their capabilities and contribute meaningfully to society.

## Figures and Tables

**Table 1:** Background information of participants.

Participant	Cohort	Gender	Current Occupation/Education
S1	2	Male	Music creator; text editor
S2	2	Female	Lawyer
S3	4	Male	R&D engineer
S4	5	Female	Graduate Institute of Law
S5	4	Female	Social worker
S6	4	Male	Teacher
S7	4	Female	Seminary student
S8	4	Male	M.A. student, History
S9	4	Female	Advertising director; executive producer/director
S10	4	Male	Ph. D. student, Earth Science
S11	6	Male	Junior (3rd year), University of Education
S12	6	Female	Junior (3rd year), Economics
S13	5	Female	5th-year student, education-related program
S14	1	Female	Lawyer
S15	4	Female	Electronics company; product marketing
S16	5	Male	Senior (4th year), German
S17	4	Female	Graduate Institute of Law
S18	6	Female	Junior (3rd year), Psychology
S19	3	Female	Table tennis coach; digital illustration
S20	1	Female	Early childhood education teacher
S21	2	Female	Supervisor, building-materials/hardware store
S22	2	Female	Writer
S23	2	Female	Bank clerk
S24	2	Male	Engineer
S25	1	Female	Accountant

## Data Availability

The data presented in this study are available on request from the corresponding author. The data are not publicly available due to privacy.
